# Low fasting glucose is associated with enhanced thrombin generation and unfavorable fibrin clot properties in type 2 diabetic patients with high cardiovascular risk

**DOI:** 10.1186/s12933-015-0207-2

**Published:** 2015-05-01

**Authors:** Grzegorz Gajos, Malgorzata Konieczynska, Jaroslaw Zalewski, Anetta Undas

**Affiliations:** Department of Coronary Disease and Heart Failure, Institute of Cardiology, Jagiellonian University Medical College, 80 Pradnicka str, 31-202 Krakow, Poland; John Paul II Hospital, Krakow, Poland; Institute of Cardiology, Jagiellonian University Medical College, Krakow, Poland

**Keywords:** Blood glucose, Cardiovascular diseases, Diabetes complications, Fibrin, Hypoglycemia

## Abstract

**Objective:**

To investigate the effect of low blood glucose on thrombin generation and fibrin clot properties in type 2 diabetes (T2DM).

**Methods:**

In 165 patients with T2DM and high cardiovascular risk, we measured ex vivo plasma fibrin clot permeation [K_s_], turbidity and efficiency of fibrinolysis including clot lysis time [t_50%_], together with thrombin generation and platelet activation markers in relation to fasting blood glucose.

**Results:**

As compared to patients in medium (4.5-6.0 mmol/l, n = 52) and higher (>6.0 mmol/l, n = 75) glucose group, subjects with low glycemia (<4.5 mmol/l, n = 38) had lower K_s_ by 11% (p < 0.001) and 8% (p = 0.01), respectively, prolonged t_50%_ by 10% (p < 0.001) and 7% (p = 0.016), respectively, and higher peak thrombin generation by 21% and 16%, respectively (p < 0.001 for both). There were no significant differences in K_s_ and t_50%_ between patients in medium and higher glucose group. In the whole group, a J-shape relationship was observed between glycemia and the following factors: peak thrombin generation, K_s_ and t_50%_. Only in patients with HbA1c < 6.0% (42 mmol/mol) (n = 26) fasting glucose positively correlated with K_s_ (r = 0.53, P = 0.006) and inversely with t_50%_ (r = −0.46, P = 0.02).

By multiple regression analysis, after adjustment for age, fibrinogen, HbA1c, insulin treatment and T2DM duration, fasting glycemia was the independent predictor of K_s_ (F = 6.6, df = 2, P = 0.002), t_50%_ (F = 8.0, df = 2, P < 0.001) and peak thrombin generation (F = 13.5, df = 2, P < 0.0001).

**Conclusions:**

In T2DM patients fasting glycemia <4.5 mmol/l is associated with enhanced thrombin formation and formation of denser fibrin clots displaying lower lysability, especially when strict glycemia control was achieved (HbA1c<6.0%).

## Background

Patients with type 2 diabetes who are at high risk of cardiovascular (CV) events have increased morbidity and mortality. The optimal treatment of hyperglycemia in those patients is not yet established [1]. Surprisingly, recent clinical trials have clearly demonstrated that intensive glucose lowering treatment provides limited benefits on CV and all cause mortality in patients with type 2 diabetes and CV disease [1] or might be even harmful [1]. Treatment-related hypoglycemia has been identified as a potential factor for worsening the prognosis in diabetes [2,3]. However, mechanisms underlying limited benefits from intensive glucose lowering in type 2 diabetes patients with high CV risk are unclear. They involve adrenergic activation [3], combined with several atherogenic and pro-inflammatory effects [4,5]. It is known that even mild asymptomatic hypoglycemia (approximately 58–69 mg/dL [3.2-3.8 mmol/L]) stimulates counter regulatory hormones i.e. adrenaline, noradrenaline and may adversely affect the prognosis not only in patients with type 2 diabetes [3].

Indeed, in large Diabetes Epidemiology: Collaborative Analysis Of Diagnostic Criteria in Europe (DECODE) study it was shown that the relation between mortality and fasting blood glucose (FBG) concentration followed a J-shaped curve both in people without diabetes and in diabetic patients [6]. Compared with an FPG of 4.50–6.09 mmol/l, subjects with FPG <4.50 mmol/l had higher both cardiovascular and all-cause mortality [6]. Moreover, it has also been recently demonstrated in the analysis by the Emerging Risk Factors Collaboration that in a large number of prospective studies of participants without diabetes the J-shaped associations exist between various glycemia measures (including FBG) and CV risk [7].

Unfortunately, the knowledge of real incidence of hypoglycemia or low glycemia in large clinical trials and its clinical significance is limited, as they rely mainly on symptomatic and self-reported episodes. It has been suggested that the harm associated with repeated episodes of hypoglycemia might counterbalance the potential benefit of intensive glucose lowering treatment [8].

Several lines of evidence indicate that hyperglycemia in type 2 diabetes results in several prothrombotic changes, including unfavorable fibrin clot structure. Dunn et al. [9] showed that clots formed by fibrinogen purified from type 2 diabetes subjects free of clinically overt CV disease had a denser, less porous structure than those from control subjects. Both low (<5 mmol/l) and high glucose concentrations (>10 mmol/l) were associated with unfavorable structural changes in fibrin network that were largely driven by fibrinogen glycation [9]. Resistance of fibrin clots to lysis in type 2 diabetes may also result from decreased binding of tissue plasminogen activator (t-PA) and plasminogen to fibrin, increased α2-antiplasmin cross-linking, [10] and increased glycation of plasminogen leading to lower plasmin generation and protein-specific activity [11]. Several studies demonstrated that enhanced platelet activation and thrombin generation, together with altered fibrin clot properties, could increase the risk of myocardial infarction and stroke in type 2 diabetes [12-14]. Recently, it has been shown that prolonged duration of type 2 diabetes, on top of inadequate glycemia control, is associated with increased thrombin formation and prothrombotic fibrin clot phenotype [15]. Additionally, stable angina, previous myocardial infarction (MI) or stroke, arterial hypertension, current smoking and family history of MI adversely affect fibrin clot formation and degradation [16,17].

To date, few studies have investigated the effects of hypoglycemia on blood coagulation. It was previously shown that insulin-induced severe hypoglycemia in healthy subjects promotes platelet aggregation [18,19]. Adrenergic stimulation and plasma epinephrine release were proven to be major determinants of platelet activation in insulin-induced severe hypoglycemia in type 2 diabetes [20]. Enhanced thrombin generation and increased plasma factor VIII activity induced by hypoglycemia were observed in type 1 diabetes [21]. Higher plasma levels of von Willebrand factor with increased blood viscosity have been documented in response to insulin-induced hypoglycemia without any changes in fibrinogen or fibrin degradation products [22]. Most of those studies explored hemostatic effects of symptomatic insulin-induced severe hypoglycemia. Of note, in a recent observation in type 2 diabetes patients severe hypoglycemia induced during hyperinsulinemic clamp study provoked prolonged (for at least 1 week) prothrombotic changes in the fibrin network and enhanced inflammation, for at least 1 week [23]. Little is known about alterations to blood coagulation and fibrin clot properties in mildly decreased glycemia in type 2 diabetes, especially in patients with high cardiovascular risk.

Given the paucity of data on the effects of hypoglycemia on blood coagulation, we tested the hypothesis that low blood glucose, like hyperglycemia, unfavorably alters thrombin generation, platelet activation and fibrin clot properties in patients with type 2 diabetes and high CV risk.

## Materials and methods

We screened 255 consecutive stable patients with type 2 diabetes that was diagnosed according to the American Diabetes Association criteria [24]. Only patients with high CV risk defined as the presence of either atherosclerotic CV disease or 2 concomitant risk factors (arterial hypertension, hyperlipidemia, obesity, current smoking, family history of CV) were included. The exclusion criteria were as follows: arterial or venous thromboembolic events within the previous 6 months, current anticoagulant or heparin therapy, known cancer, chronic inflammatory disease, liver injury (alanine and asparagine transaminase >1.5 times above the upper limit of the reference range), glomerular filtration rate (GFR) <30 mL/min, pregnancy, all the states known to alter blood coagulation and/or fibrin clot structure [14,16].

Arterial hypertension was defined as a systolic and/or a diastolic blood pressure measurement consistently ≥140 mmHg or ≥90 mmHg, respectively. Dyslipidemia was defined as total cholesterol (TC) >5.0 mmol/L and low-density lipoprotein (LDL) cholesterol >2.6 mmol/L or ongoing lipid-lowering treatment. Obesity was diagnosed when BMI ≥30 kg/m^2^. A positive family history was defined as evidence of coronary artery disease (CAD) in a first degree relative in men before the age of 55 years and women before the age of 65 years. CAD was established based on documented history of myocardial infarction (MI) or positive results of ECG stress test or gated single photon emission computed tomography with Tc-99 m-MIBI (SPECT) or coronary angiography. Peripheral artery disease was defined based on ankle-brachial index (ABI) <0.9. Previous MI, ischemic stroke or previous revascularization was established based on medical records. Diabetic nephropathy (DN) was defined as macroalbuminuria (albumin to creatinine ratio [ACR] >34 mg/mmol [300 mg/g]), or microalbuminuria (ACR 3.4 to 34 mg/mmol [30 to 300 mg/g]) associated with retinopathy. Diabetic neuropathy was diagnosed according to the Toronto Diabetic Neuropathy Expert Group definition [25]. An experienced ophthalmologist diagnosed diabetic retinopathy.

The study was powered to have an 90% chance of detecting a 10% difference in plasma clot permeability between patents with asymptomatic low (<4.5 mmol/L) and normal (>4.5 mmol/L) FBG using a *P* value of 0.05, based on the values of fibrin features in the published article [26]. In order to demonstrate such a difference or greater, 30 patients were required in each group. For a *P* value of 0.01, 43 patients per group were required.

The local ethics committee approved the study. All subjects enrolled provided written, informed consent.

### Laboratory investigations

Fasting blood samples were drawn between 8:00 and 10:00 AM from an antecubital vein. Plasma samples (9:1 of 3.2% trisodium citrate) for the hemostasis assay were centrifuged (20 minutes, 2500 g) within 30 minutes of collection, immediately frozen, and stored in aliquots at −80°C. Routine blood tests, including glucose (the hexokinase method), lipid profile, blood cell count, transaminases, and serum creatinine were carried out by automated laboratory techniques.

According to FBG patients were divided into 3 groups: with LOWER glucose (<4.5 mmol/l), with MEDIUM glucose (4.5-6.0 mmol/l) and with HIGHER glucose (>6.0 mmol/l). The lower cut-off values was chosen arbitrarily, but there were based on the data from DECODE study demonstrating that subjects with FBG <4.50 mmol/l in comparison to FBG 4.50–6.09 mmol/l had higher cardiovascular and total mortality in the follow-up [6].

HbA_1C_ and high sensitivity C-reactive protein (CRP) were measured using immunoturbidimetric assays (Roche Diagnostics GmbH, Mannheim, Germany, Tina-quant Hemoglobin A_1c_ Gen.2 and Cardiac C-Reactive Protein [Latex] High Sensitive, respectively). Fibrinogen was determined with the von Clauss method. Plasminogen and antiplasmin were measured by chromogenic assays (STA Stachrom plasminogen and STA Stachrom α2-antiplasmin, Diagnostica Stago, Asniéres, France). Using commercially available enzyme-linked immunoabsorbent assays, we determined in plasma PAI-1 antigen (PAI-1: Ag) (American Diagnostica, Stamford, CT, USA), thrombin-activatable fibrinolysis inhibitor (TAFI) antigen (Chromogenix, Lexington, MA, USA) and plasma markers of platelet activation: soluble CD_40_ ligand (sCD40L), platelet factor-4 (PF4) (R@D systems). The interassay and intraassay coefficients of variation for all the ELISAs were <8%.

#### Thrombin generation potential

To assess plasma thrombogenic potential, the thrombogram was analyzed using the CAT (Thrombinoscope BV, Maastricht, the Netherlands) according to the manufacturer's instructions in the 96-well plate fluorometer (Ascent Reader, Thermolabsystems OY, Helsinki, Finland) equipped with the 390/460 filter set at a temperature of 37°C [15]. Each plasma sample was analyzed in duplicate, and the intraassay variability was 6%. The peak thrombin level was analyzed.

#### Fibrin clot permeability

Fibrin clot permeation properties were determined as previously described [26,27]. A permeation coefficient (K_s_), which indicates the size of fibrin clot pores, was calculated from the following equation: K_s_ = Q × L × η/t × A × Δp, where Q is the flow rate in time t, L is the length of a fibrin gel (13 mm), η is the viscosity of the liquid (1/100 poise), A is a cross-sectional area (0.049 cm^2^), and Δp is a differential pressure (in dyne/cm^2^). The interassay and intraassay coefficients of variation were <9% (n = 20).

#### Plasma clot lysis assays

Plasmin-mediated fibrinolysis in the presence of recombinant tissue plasminogen activator (Boehringer Ingelheim, Ingelheim, Germany) was evaluated as previously described [26,27]. Lysis time was defined as the time required for a 50% decrease in fibrin clot absorbance (t_50%_) and was chosen as a marker of the clot susceptibility to fibrinolysis. The interassay and intraassay coefficients of variation were below <8%.

In the second assay, fibrin clots, formed as described above, were perfused with the same buffer containing 0.2 μmol L-1 rt-PA according to Collet et al. [28]. The lysis rate was determined by measuring the concentration of D-dimers (American Diagnostica), a marker of plasmin-mediated fibrin degradation, in the effluent every 20 min. The maximum rate of increase in D-dimer levels (D-Drate, mg/L/min) and maximum D-dimer concentrations (D-Dmax) were determined in each subject (intraindividual variability, 8%). The experiment was stopped usually after 80–120 min when the fibrin gel collapsed under the pressure.

#### Turbidity measurements

We determined the lag phase, which reflects the time required for fibrin protofibrils to grow to sufficient length to allow lateral aggregation and maximum absorbance at plateau (ΔAbs_max_), which reflects the number of protofibrils per fiber, as previously described [29]. The coefficients of interassay and intraassay variations were from 5.5 to 7.2%.

### Statistical analysis

Statistical analyses were performed with SPSS 20.0 software. Continuous variables are expressed as mean ± SD or median (interquartile range) and categorical variables as number (percentage). Continuous variables were first checked for normal distribution by the Shapiro-Wilk statistic. Analysis of variance followed by Bonferroni test was used to compare differences in the three groups with normally distributed data whereas non-normally distributed data were analyzed by Kruskal-Wallis test and differences between groups were identified using a test for multiple comparisons of mean rank. Categorical variables were compared by chi-square test. The J-shape relationship between fibrin clot permeability (y_1_), lysis time (y_2_), peak thrombin generation (y_3_) versus fasting bood glucose concentration (x) was evaluated on the basis of generalized linear model. The studied relationships were best fitted with quadratic polynomial equation (y = β_0_ + β_1_ × x + β_2_ × x^2^). The Pearson or Spearman rank correlation coefficients were calculated to test the association between two variables with a normal or non-normal distribution, respectively. Analysis of covariance assuming homogeneity of slopes was used to compare fibrin clot properties and thrombin generation in the study groups with adjustment for age, fibrinogen and diabetes duration. All clinical, laboratory and angiographic variables that showed the association with clot permeability or lysis time in univariate model and did not show significant correlations with another independent variable were then included in the multiple regression analysis to determine predictors of fibrin clot properties and thrombin generation. A two-sided *P* < 0.05 was considered statistically significant.

## Results

One hundred sixty five patients with type 2 diabetes were included and 38 had low FBG levels, with no severe and symptomatic hypoglycemia in the LOWER glucose group (median 4.0 mmol/l; IQR 3.6-4.2) (Table 1). The three groups did not differ in terms of demographic variables, cardiovascular risk factors, medications and baseline laboratory investigations except HbA1 level that was lower and insulin use which was lower in MEDIUM glucose group in comparison with other groups (Table 1). Patients in the LOWER glucose group showed a nonsignificant trend to higher hematocrit values and to a higher prevalence of nephro- and neuropathy than individuals in the remaining groups (Table 1).

**Table 1:** **Baseline characteristics and laboratory investigations**

	**LOWER glucose group (<4.5 mmol/l) N = 38**	**MEDIUM glucose group (4.5-6.0 mmol/l) N = 52**	**HIGHER glucose group (>6.0 mmol/l) N = 75**	**P-value**
Female, n (%)	15 (39.5)	27 (51.9)	30 (40.0)	0.35
Age, yrs	67 (59–72)	68 (63–73)	66 (59–73)	0.63
Weight, kg	86 (75–100)	84 (75–92)	86 (78–95)	0.24
Height, cm	169 (163–175)	165 (158–170)	168 (158–173)	0.11
BMI, kg/m^2^	30.8 (26.1-33.7)	31.4 (27.5-35.8)	31.9 (28.4-36.1)	0.17
Waist, cm	107 (93–112)	106 (98–112)	107 (99–113)	0.90
Hip, cm	109 (99–116)	109 (102–115)	109 (104–116)	0.85
Duration of DM, yrs	8 (3–10)	5 (3–7)	5 (3–11)	0.10
CAD, n (%)	24 (63.2)	33 (63.5)	43 (57.3)	0.74
Previous MI, n (%)	9 (23.7)	7 (13.5)	10 (13.3)	0.31
Hypertension, n (%)	35 (92.1)	49 (94.2)	74 (98.7)	0.21
Current smoking, n (%)	4 (10.5)	3 (5.8)	7 (9.3)	0.69
History of smoking, n (%)	17 (44.7)	25 (48.1)	36 (48.0)	0.94
Family history of CAD, n (%)	15 (39.5)	13 (25.0)	16 (21.3)	0.11
Retinopathy, n (%)	4 (10.5)	7 (13.5)	13 (17.3)	0.61
Nephropathy, n (%)	10 (26.3)	4 (7.7)	14 (18.7)	0.06
Neuropathy, n (%)	13 (34.2)	9 (17.3)	13 (17.3)	0.08
**Pharmacotherapy**				
Sulphonylourea, n (%)	17 (44.7)	23 (44.2)	30 (40.0)	0.85
Biguanide, n (%)	23 (60.5)	35 (67.3)	48 (64.0)	0.80
Insulin therapy, n (%)	15 (39.5)	5 (9.6)	24 (32.0)	0.002
Beta-blocker, n (%)	26 (68.4)	39 (75.0)	63 (84.0)	0.15
ACEI, n (%)	30 (78.9)	35 (67.3)	56 (74.7)	0.44
Calcium antagonist, n (%)	14 (36.8)	21 (40.4)	25 (33.3)	0.72
Clopidogrel, n (%)	1 (2.6)	4 (7.7)	4 (5.3)	0.58
Aspirin, n (%)	33 (86.8)	39 (75.0)	61 (81.3)	0.37
Statin, n (%)	35 (92.1)	43 (82.7)	57 (76.0)	0.11
**Laboratory results**				
FBG mmol/l	4 (3.6-4.2)	5.35 (4.9-5.7)	7.3 (6.4-8.3)	<0.0001
HbA1c, %	6.7 (6.1-7.3)	6.2 (6.0-6.7)	6.8 (6.3-7.6)	0.0002
HbA1c, mmol/mol	50 (43–56)	44 (42–50)	51 (45–60)	0.0002
WBC, x10^9^/L	6.5 (5.2-7.9)	7.1 (6.2-8.2)	7.3 (6.2-7.9)	0.23
Hemoglobin, g/dL	13.5 (12.9-14.5)	13.8 (13.2-14.4)	13.7 (13.0-14.6)	0.64
Hematocrit, %	43.0 (39.0-46.0)	41.7 (39.9-43.3)	41.2 (39.1-43.2)	0.07
Platelet count, x10^9^/L	227 (176–252)	206 (172–252)	216 (186–266)	0.61
INR	0.99 (0.96-1.08)	0.98 (0.95-1.03)	0.98 (0.94-1.02)	0.18
Creatinine, μmol/L	76 (66–91)	81 (63–92)	79 (66–98)	0.56
Alat, IU/L	21 (12–27)	20 (13–29)	22 (16–30)	0.10
Aspat, IU/L	24 (18–28)	20 (16–25)	20 (18–26)	0.62
TC, mmol/L	4.17 (3.42-4.98)	4.20 (3.51-5.04)	4.43 (3.68-5.32)	0.10
LDL-C cholesterol, mmol/L	2.20 (1.71-2.80)	2.26 (1.92-3.04)	2.57 (2.06-3.41)	0.16
HDL-C cholesterol, mmol/L	1.11 (0.88-1.35)	1.40 (1.18-1.62)	1.35 (1.08-1.59)	0.08
TG, mmol/L	1.16 (0.94-1.80)	1.31 (0.92-1.56)	1.48 (1.01-1.96)	0.37
hsCRP, mg/dl	2.57 (0.89-5.01)	1.85 (1.02-3.21)	2.29 (1.04-5.08)	0.31

Values are given as mean ± SD, median (interquartile range).

*Abbreviations:* ACEI, angiotensin converting enzyme inhibitor; Alat, alanine transaminase; Aspat, aspartate transaminase; BMI, body mass index; CAD, coronary artery disease; FBG, fasting blood glucose; hsCRP, high sensitivity C-reactive protein; DM, diabetes mellitus; HbA1c, glycated hemoglobin; HDL-C, high-density lipoprotein cholesterol; INR, International normalized ratio; MI, myocardial infarction; LDL-C, low-density lipoprotein cholesterol; TC, total cholesterol; TG, triglycerides; WBC, white blood cells.

### Thrombin generation and platelet activation markers

**Figure 1:**
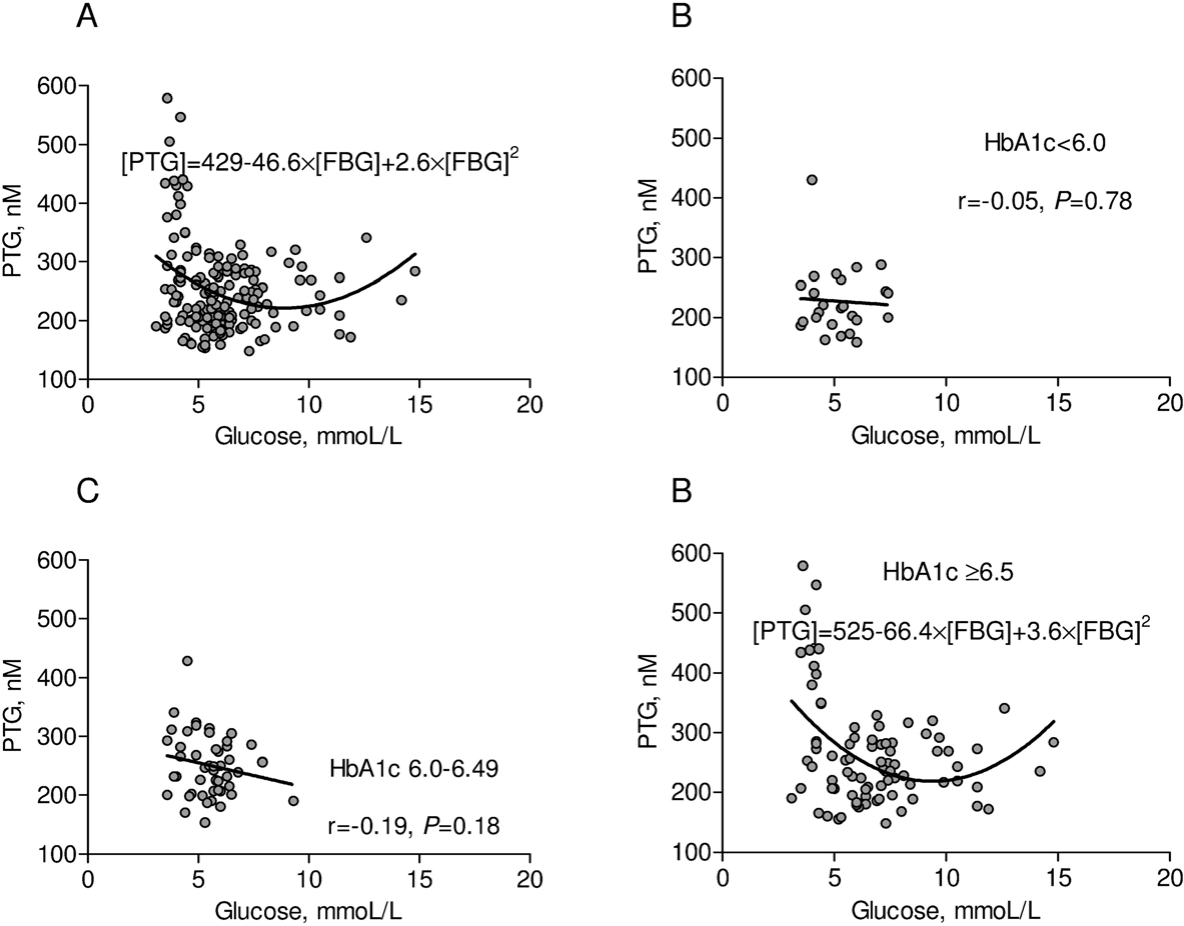
Peak thrombin generation in relation to glucose and HbA1c level. **(A)** Peak thrombin generation [PTG] in relation to fasting blood glucose [FBG] for the whole group. **(B)**-**(D)** Peak thrombin generation in relation to fasting glucose depending on the various levels of HbA1c. P-values for **(A)** and **(D)** are in the text of manuscript.

A non-linear significant J-shape relationship between glycemia and peak thrombin generation was found in the whole group (β_0_ = 429, β_1_ = −46.6, β_2_ = 2.6, p < 0.001 for each component, Figure 1A) and also in patients with HbA1c > 6.5% (β_0_ = 525, β_1_ = −66.4, β_2_ = 3.6, p < 0.001 for each component, Figure 1D). The highest peak thrombin generation was observed in patients in LOWER glucose group (p = 0.002). Those patients with low glycemia had 21.3% and 15.7% higher thrombin generation in comparison to the MEDIUM and HIGHER glucose groups, respectively (p < 0.001 for both) (Table 2).

**Table 2:** **Coagulation, platelet function, fibrinolysis and fibrin clot properties**

	**LOWER glucose group (<4.5 mmol/l)**	**MEDIUM glucose group (4.5-6.0 mmol/l)**	**HIGHER glucose group (>6.0 mmol/l)**	**P-value**
Fibrinogen, g/dL	3.06 (2.66-3.48)	3.13 (2.67-3.57)	3.15 (2.64-3.61)	0.98
Peak thrombin, nM	273 (209–380)	225 (201–268)*	236 (196–275)*	0.002
PF4	10.6 (9.1-12.8)	9.5 (8.2-12.0)	10.3 (8.2-12.8)	0.26
sCD40L	4.3 (3.6-4.9)	3.7 (3.1-4.4)	3.8 (3.3-4.8)	0.09
TAFI, %	71.5 (41.6-90.0)	91.0 (84.0-106.0)*	100.0 (92.0-111.0)*	<0.0001
PAI-1: Ag	30.6 (25.8-36.1)	32.0 (28.7-36.3)	33.9 (29.4-38.9)	0.23
Plasminogen, %	109 ± 21	108 ± 16	110 ± 14	0.83
Antiplasmin, %	109 (100–118)	109 (96–118)	107 (97–117)	0.61
K_s_, cm^2^x10^−9^	6.52 ± 0.79	7.29 ± 0.81*	7.06 ± 0.88*	0.0001
t_50%_, min	10.49 ± 0.97	9.55 ± 0.91*	9.79 ± 1.11*	<0.0001
Lag_phase, s	45 (41–50)	44 (41–47.0)	43 (40–45)*	0.019
ΔAbs_max_, 405 nm	0.79 (0.76-0.83)	0.81 (0.77-0.86)	0.82 (0.80-0.87)*	0.015
D-D_rate_ mg/L/min	0.069 ± 0.006	0.070 ± 0.005	0.07 ± 0.004	0.47
D-D_max_ mg/L	3.90 (3.78-4.22)	3.87 (3.69-4.06)	3.88 (3.62-4.22)	0.30

Values are given as mean ± SD, median (interquartile range). *P* value was measured using analysis of variance (ANOVA) with post-hoc Bonferroni test or Kruskal-Wallis test with multiple comparisons of mean rank, *p < 0.05 as compared with LOWER glucose group.

*Abbreviations:* ΔAbs_max_, maximum absorbance of fibrin gel at 405 nm determined by using turbidimetry; sCD40L, soluble CD_40_ ligand; D-D_max_, maximum D-dimer levels in the lysis assay; D-D_rate_, maximum rate of increase in D-dimer levels in the lysis assay; K_s_, permeability coefficient; PAI-1: Ag; plasminogen activator inhibitor-1 antigen; PF4, platelet factor 4; t_50%_, half-lysis time; TAFI, thrombin-activatable fibrinolysis inhibitor.

Plasma PF4 levels were similar in all the three groups (Table 2). There was a trend towards higher sCD40L in patients with LOWER glucose as compared to normo- and hyperglycemic patients (Table 2).

### Fibrinolytic proteins

Plasminogen and antiplasmin levels were unaffected by FBG levels. Interestingly, the highest TAFI levels were observed in patients in the HIGHER glucose group. They were 40% and 10% higher than in patients with LOWER and MEDIUM glucose, respectively (both p < 0.0001).

### Fibrin clot formation, porosity and lysis

**Figure 2:**
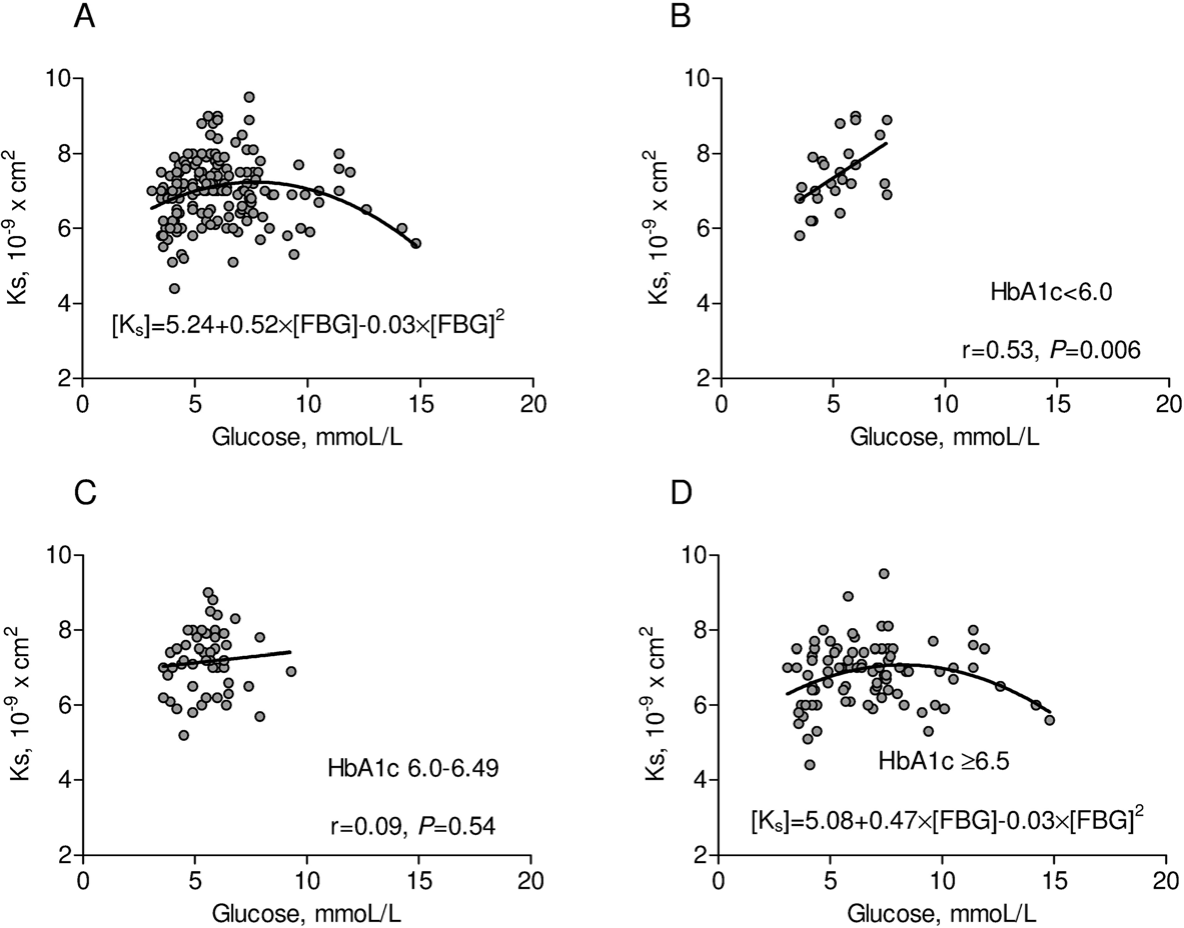
Clot permeability in relation to glucose and HbA1c level. **(A)** Clot permeability [Ks] in relation to fasting blood glucose [FBG] for the whole group. **(B)**-**(D)** Ks in relation to fasting glucose depending on the various levels of HbA1c. P-values for **(A)** and **(D)** are in the text of manuscript.

**Figure 3:**
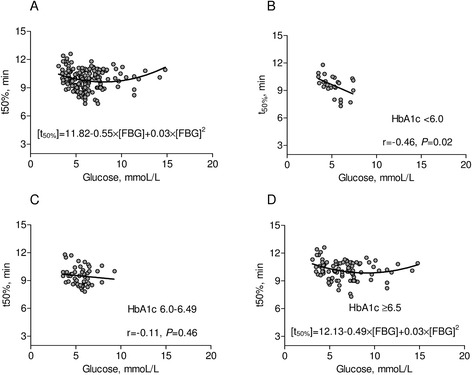
Clot lysis time in relation to FBG and HbA1c level. **(A)** Lysis time [t50%] in relation to fasting glucose for the whole group. **(B)**-**(D)** t50% in relation to fasting blood glucose [FBG] depending on the various levels of HbA1c. P-values for **(A)** and **(D)** are in the text of manuscript.

**Figure 4:**
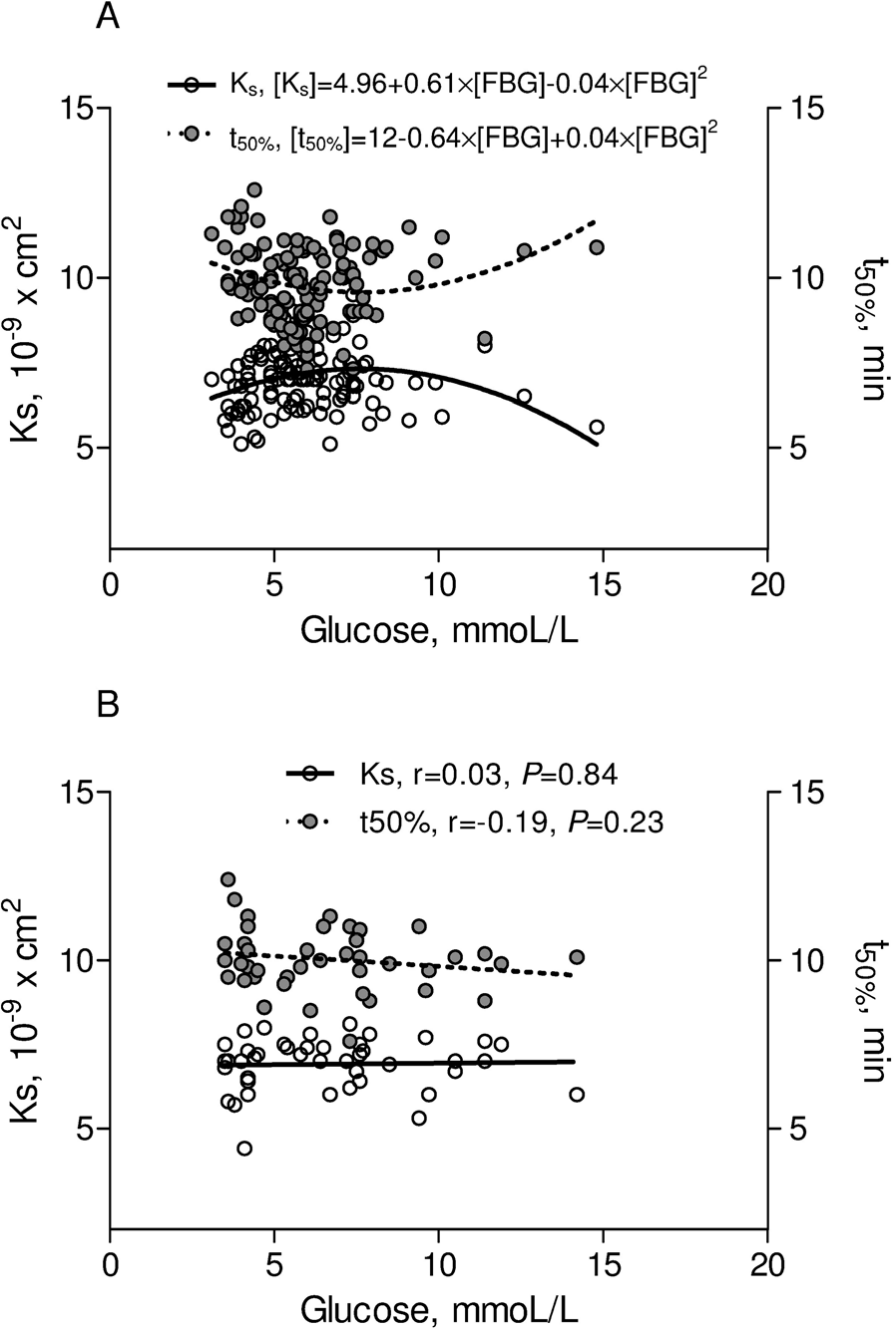
Ks and t50% versus glucose, according to insulin treatment. T2DM patients without **(A)** and with **(B)** insulin treatment. P-values for **(A)** are in the text of manuscript. Abbreviations: as Figures 1 and 2.

Interestingly, we observed also a J-shape relationship between FBG levels and both fibrin clot permeability (β_0_ = 5.24, β_1_ = 0.52, β_2_ = −0.03, p < 0.001 for each component, Figure 2A) and lysis time (β_0_ = 11.82, p < 0.001; β_1_ = −0.55, p = 0.003; β_2_ = 0.03, p = 0.004, Figure 3A). The highest K_s_ and the shortest t_50%_ were found in the MEDIUM glucose group (Table 2). As compared to patients from the latter group, K_s_ was lower by 11% (p < 0.001) and t_50%_ was prolonged by 10% in patients with LOWER glucose (p < 0.001) (Table 2). In contrast, there were no differences in fibrin clot permeability and lysis time between MEDIUM glucose group and patients with HIGHER glucose values. There was a significant J-shape relationship betwen fasting bood glucose versus K_s_ (β_0_ = 4.96, p < 0.001; β_1_ = 0.61, p = 0.002; β_2_ = −0.04, p = 0.002) and t_50_ (β_0_ = 12.0, p < 0.001; β_1_ = −0.64, p = 0.008; β_2_ = 0.04, p = 0.007) in T2DM patients treated without insulin (Figure 4A) but not on insulin (Figure 4B).

In patients with HbA1c > 6.5% significant J-shape relationships between FBG versus K_s_ (β_0_ = 5.08, p < 0.001; β_1_ = 0.47, p = 0.006; β_2_ = −0.03, p = 0.007; Figure 2D) and t_50%_ (β_0_ = 12.1, p < 0.001; β_1_ = −0.49, p = 0.018; β_2_ = 0.03, p = 0.036; Figure 3D) have been found. In patients with HbA1c < 6.0% (42 mmol/l), there was a positive linear correlation between glycemia and K_s_ (r = 0.53, p = 0.006, Figure 1B) and an inverse correlation between glycemia and t_50%_ (r = −0.46, p = 0.02, Figure 2B). In the LOWER glucose group we observed slightly prolonged lag phase and lower ΔAbs_max_ compared with other patients. D-D_rate_ and D-D_max_ were similar in the three groups (Table 2).

### Correlations

**Figure 5:**
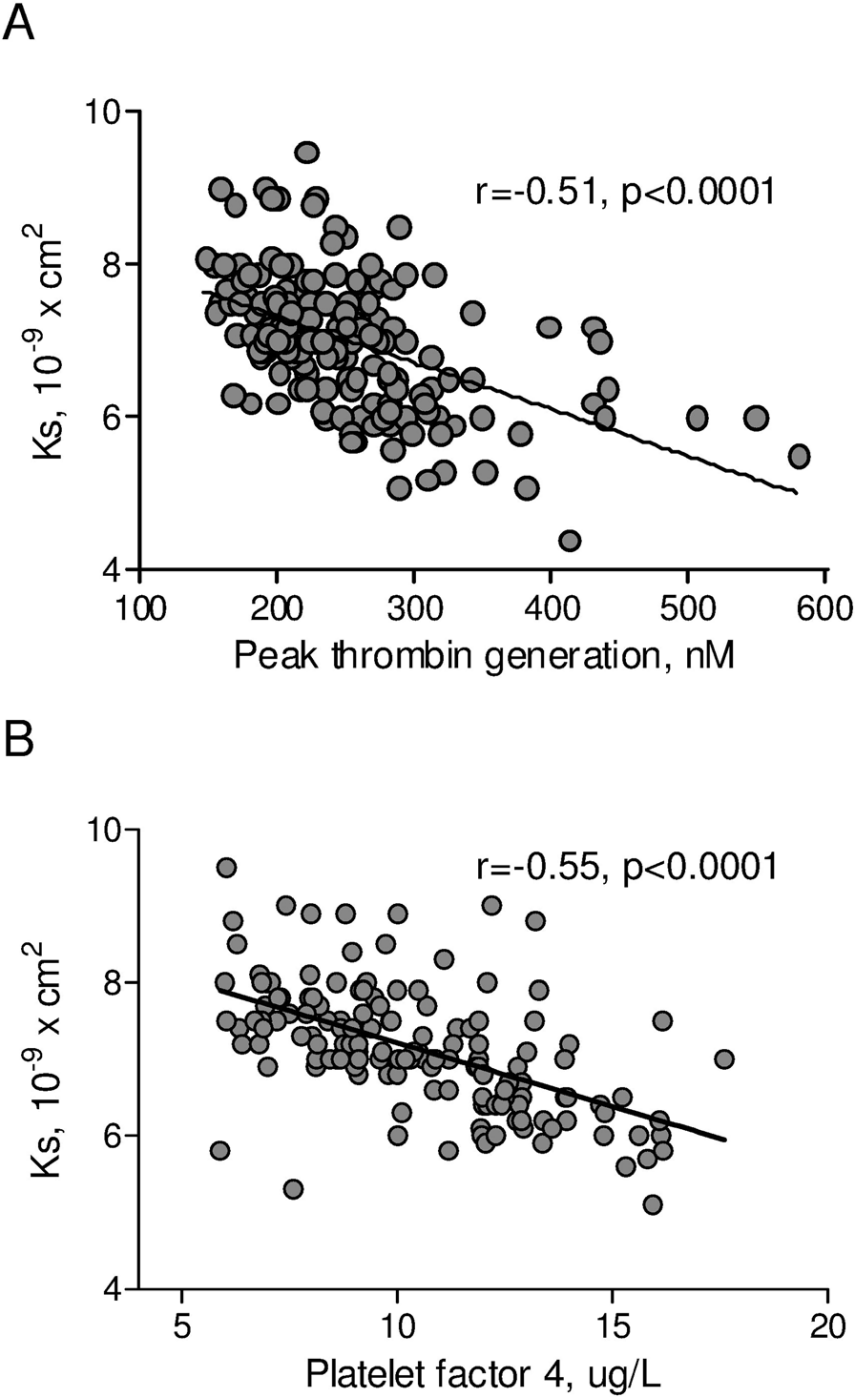
The relationships between clot permeability and thrombin generation and platelet function. **(A)** K_s_ and peak thrombin generation. **(B)** K_s_ and platelet factor-4 (PF4). Abbreviations: as Table 1 and Figure 1.

In the whole group both peak thrombin generation and PF4 were inversely correlated with K_s_ (r = −0.51, p < 0001 and r = −0.55, p < 0.0001 respectively, Figure 5). Moreover, peak thrombin generation was negatively correlated with D-D_rate_, TAFI and plasminogen and positively correlated with t_50%_ and D-D_max_ (Table 3). Simultaneously, PF4 and sCD40L were inversely correlated with D-D_rate_ and positively correlated with t_50%_, D-D_max_ and ΔAbs_max_ (Table 3).

**Table 3:** **Fibrin clot properties in relation to fibrinogen, platelet function and thrombin generation**

	**Fibrinogen**	**Peak thrombin**	**PF4**	**sCD40L**
K_s_	r = −0.34	r = −0.51	r = −0.55	r = −0.37
	p < 0.0001	p < 0.0001	p < 0.0001	p < 0.0001
t_50%_	r = 0.32	r = 0.41	r = 0.41	r = 0.37
	p < 0.0001	p < 0.0001	p < 0.0001	p < 0.0001
D-D_max_	r = 0.33	r = 0.21	r = 0.17	r = 0.23
	p < 0.0001	p = 0.007	p = 0.031	p = 0.003
D-D_rate_	r = −0.53	r = −0.25	r = −0.15	r = −0.18
	p < 0.0001	p = 0.001	p = 0.056	p = 0.019
lag phase	r = −0.01	r = 0.16	r = −0.05	r = −0.06
	p = 0.85	p = 0.035	p = 0.54	p = 0.48
ΔAbs_max_	r = 0.16	r = −0.04	r = 0.23	r = 0.11
	p = 0.044	p = 0.63	p = 0.003	p = 0.14
TAFI	r = −0.05	r = −0.51	r = −0.04	r = −0.17
	p = 0.56	p < 0.0001	p = 0.64	p = 0.037
Plasminogen	r = −0.13	r = −0.22	r = 0.07	r = 0.12
	p = 0.11	p = 0.005	p = 0.36	p = 0.15
Antiplasmin	r = −0.27	r = −0.06	r = 0.01	r = −0.01
	p = 0.001	p = 0.47	p = 0.89	p = 0.97

*Abbreviations:* as in Tables 1 and 2.

### Independent predictors of fibrin clot phenotype

The multiple regression analysis was used to determine independent predictors of fibrin clot properties and peak thrombin genertion (Table 4). Before the inclusion to the multiple analysis model, independent variables including age, gender, duration of diabetes mellitus, height, hip measurement, treatment with aspirin, HbA1c, platelet count, fibrinogen, glucose concentration and glucose concentration squared, PF4 and CD40L were associated in an univariate model with clot permeability. Gender, duration of diabetes mellitus, hip measurement, treatment with aspirin, HbA1c level, fibrinogen, triglycerides, glucose concentration and glucose concentration squared, PF4 and CD40L were associated with t_50%_. In turn, age, duration of diabetes mellitus, treatment with aspirin, HbA1c, fibrinogen, glucose concentration and glucose concentration squared were associated with peak thrombin generation.

**Table 4:** **The multiple regression analysis with clot permeability and lysis time as the dependent variables**

**Dependent variable**	**Independent variables**	***P*** **-value**	**Coefficient**	**95.0% CI**	
Clot permeability	PF4	<0.001	−0.46	−0.57	−0.33
R = 0.62, R^2^ = 0.38,	Fibrinogen	<0.001	−0.27	−0.41	−0.12
F(6, 156) = 13.8, p < 0.0001	HbA1c	0.002	−0.19	−0.33	−0.04
	DM duration	0.005	−0.22	−0.36	−0.07
	Glucose	0.002	0.61	0.50	0.70
	Glucose squared	0.008	−0.56	−0.66	−0.44
Lysis time	PF4	<0.001	0.41	0.27	0.53
R = 0.58, R^2^ = 0.34,	Fibrinogen	<0.001	0.22	0.07	0.36
F(6, 156) = 11.4, P < 0.0001	DM duration	0.002	0.25	0.10	0.39
	HbA1c	0.002	0.20	0.05	0.34
	Glucose	0.008	−0.47	−0.58	−0.32
	Glucose squared	0.045	0.30	0.15	0.43
Peak thrombin generation	Glucose	<0.001	−0.97	−0.98	−0.96
R = 0.51, R^2^ = 0.26,	Glucose squared	0.014	0.83	0.77	0.87
F(6, 155) = 8.8, P < 0.0001	Fibrinogen	0.002	0.22	0.07	0.36
	DM duration	0.005	0.21	0.06	0.35
	HbA1c	0.015	0.21	0.06	0.36
	Age	0.03	−0.15	−0.30	0

*Abbreviations:* as in Tables 1 and 2.

After adjustment for fibrinogen (F = 20.9, df = 1, P < 0.0001), HbA1c (F = 11.2, df = 1, P = 0.001), T2DM duration (F = 8.4, df = 1, P = 0.004) and insulin therapy (F = 1.8, df = 1, P = 0.18), glucose concentration significantly influenced clot permeability (F = 6.6, df = 2, P = 0.002). By multiple analysis, the higher level of fibrinogen, HbA1c or PF4, and the longer time of DM, the more dense fibrin network. Also both low and high glucose concentrations were associated with lower clot permeability (Table 4, Figure 2A).

After adjustment for fibrinogen (F = 18.1, df = 1, P < 0.0001), HbA1c (F = 9.5, df = 1, P = 0.003), T2DM duration (F = 10.5, df = 1, P = 0.001) and insulin therapy (F = 1.6, df = 1, P = 0.21), glucose concentration significantly influenced clot lysis time (F = 8.0, df = 2, P = 0.0005). By multiple analysis, the higher level of fibrinogen, HbA1c or PF4, and the longer time of DM, the longer time of clot lysis. Also both low and high glucose concentrations were associated with prolonged lysis time (Table 4, Figure 3A).

After adjustment for fibrinogen (F = 9.0, df = 1, P = 0.003), HbA1c (F = 3.2, df = 1, P = 0.08), T2DM duration (F = 6.7, df = 1, P = 0.01) and insulin therapy (F = 2.2, df = 1, P = 0.14), glucose concentration significantly influenced peak thrombin generation (F = 13.5, df = 2, P < 0.0001). By multiple analysis, the younger patient, the higher level of fibrinogen, HbA1c, and the longer time of DM, the higher peak thrombin generation. Also both low and high glucose concentrations were associated with high peak thrombin generation (Table 4, Figure 1A).

## Discussion

### Fibrin clot properties

The current study demonstrates that even asymptomatic mild hypoglycemia in type 2 diabetes patients with high CV risk is associated with unfavorably altered plasma fibrin clot structure and lysability. We found that type 2 diabetes patients with FBG levels below 4.5 mmol/l tended to form more compact plasma clots displaying relative resistance to lysis as compared to those with higher glycemia. This held true also after adjustment for fibrinogen, HbA1c, diabetes duration and insulin treatment. Interestingly, fibrin clot properties in type 2 diabetes patients demonstrate a J-shape relationship to their plasma glucose. Additionally, we observed that only in subjects with HbA1c between 6.0% and 6.5% there was no negative effect of hypoglycemia on clot permeability and lysis. Moreover, we demonstrated that FBG levels below 4.5 mmol/l were also associated with enhanced thrombin generation and trend towards higher platelet activation, which might contribute to prothrombotic fibrin clot phenotype in this clinical setting. Our study is the first to demonstrate such prothrombotic effects in well-treated type 2 diabetes patients with high CV risk.

Although clot lysis time (t_50%_) was markedly prolonged in patients with low glycemia, another marker of fibrinolysis, i.e. D-D_rate_ was not affected. The former assay, in which coagulation is initiated by addition of human thrombin, together with rt-PA, appears to be more sensitive to glycemia-related alterations to fibrin properties as compared to the other approach, in which a formed plasma fibrin clot is subjected to relatively high concentrations of rt-PA. Effects of lysis time modifiers including body-mass index, and C-reactive protein [30] or decreased fibrinolysis inhibitor (TAFI) levels could also be detectable in assays in which fibrin formation is simultaneously initiated with fibrinolysis mimicking to some extent the in vivo situation. Of note, increased levels of PAI-1 and TAFI have been demonstrated to be involved in prothrombotic changes in hemostasis observed in diabetic patients [15]. However, in the current study in asymptomatic mild hypoglycemia these two fibrinolysis inhibitors appear to be of minor importance in hypofibrinolysis reported by us.

Importantly, altered plasma clot properties in patients with low blood glucose were apparent despite administration of aspirin, statins and angiotensin converting enzyme inhibitors (ACEI), which could favorably alter fibrin clot structure and function [14]. Given the fact that most of the patients studied were taking aspirin, ACEI and a statin, it might be hypothesized that the impact of low glucose on fibrin clots is potent enough to be detectable despite some beneficial effects attributed to those medications.

### Thrombin generation

Of particular importance is that in our study alterations to fibrin clot properties demonstrated in type 2 diabetes patients, particularly in hypoglycemia, were accompanied by an increase in peak thrombin generated in plasma samples, which is a novel observation. The CAT method used in our study is commonly considered the best measure of thrombin generation [31]. The peak thrombin generation was correlated with clot permeability and lysis in the current type 2 diabetes patients, which supports the concept that the magnitude of thrombin formation is a potent adverse modulator of fibrin clot characteristics in cardiovascular disease [32]. Therefore, we might assume that hypoglycemia-induced thrombin generation might largely contribute to prothrombotic effects in patients with type 2 diabetes and its complications.

The adverse effect of low blood glucose on thrombin generation and clot structure may have a significant impact on other blood cells. It has been recently shown that red blood cell (RBC) ultrastructure is altered in diabetic patients, where these cells are elongated and twist around spontaneously formed fibrin fibers [33]. Moreover, Pretorius et al. have shown that RBCs, upon addition of glucose or thrombin, lose the ability to maintain the discoid shape and undergo the deformation under the pressure of dense fibrin fiber networks [34]. Therefore, entrapping of the RBCs by denser fibrin clot that is formed when blood glucose is low may contribute to the formation of the tight fibrin clots. Moreover, a tendency towards higher hematocrit in the low glucose group in our study could also contribute to adverse fibrin properties [35].

### Platelet activation

In the current study platelet activation has also been identified as a fibrin-modifying factor that contributes to the links between low glycemia and prothrombotic state with altered clot phenotype in type 2 diabetes patients. Activated platelets release from alpha granules various proteins, e.g. PF4, which has high affinity for glycosaminoglycans and exerts pleiotropic effects in hemostasis and thrombosis by, among others, modulating the effects of heparin-like molecules [36]. Growing evidence, largely derived from in vitro studies, indicates that platelet activation reflected by increased levels of PF4 and release of polyphosphates adversely affects fibrin clot properties including formation of compact networks [14,37]. Amelot et al. [38] have demonstrated that especially PF4 binds to fibrin and profoundly transforms the structure of the resulting network. This prothrombotic mechanism may be relevant in type 2 diabetes patients with concomitant CAD, since hyperglycemia activates platelet aggregation, as shown e.g. in patients with acute myocardial infarction [39]. In our study although there was only a trend towards increased platelet activation in hypoglycemia, a significant correlation inverse correlation between PF4 and Ks was observed. Moreover, in multivariate analysis PF4 was an independent predictor of both K_s_ and t_50%_ in type 2 diabetes, which suggests that activated platelets directly contribute to unfavorable fibrin clot phenotype in this disease. We cannot exclude that this association with PF4 is in vivo enhanced by a potent and similar effect of polyphosphates on fibrin structure [37].

### Novelty

We have previously reported that prolonged duration of type 2 diabetes is associated with increased thrombin formation, hypofibrinolysis, and prothrombotic fibrin clot phenotype [15]. This study adds a novel observation that regardless of diabetes duration moderately low blood glucose is associated with increased thrombin generation and unfavorable clot properties. It is likely that tendency to form more compact and poorly lysable clots at low HbA1c mirrors a number of previous symptomatic or asymptomatic hypoglycemia episodes and shows their cumulative negative effects. This hypothesis merits further investigation. On the other hand, insulin administration that was much more frequently administered in patients with low glycemia has been shown to improve fibrin clot phenotype in diabetic subjects at low cardiovascular risk [40]. Also in our study, in patients with high CV risk treated with insulin, no adverse effects of both low and high blood glucose on fibrin clot properties could be observed.

We might speculate that our results might offer a potential explanation of the negative results of the ACCORD study in the intensive treatment arm, where the control of glycemia was strict and the frequency of hypoglycemia was markedly elevated [2]. Moreover, it might be one of the underlying mechanisms behind the observation from a recent meta-analysis of trials involving over 900 000 patients with type 2 diabetes that severe hypoglycemia is associated with a higher risk of cardiovascular disease [41]. Although moderate hypoglycemia was not taken into account in that meta-analysis, it might be hypothesized that in severe hypoglycemia prothrombotic effects observed by us are even more pronounced.

### Limitations

The study has several limitations. The size of the study population was relatively small. Although duration of type 2 diabetes was average and glucose control was adequate, it reflected a real-life diabetic population in our region. However, our findings cannot likely be extrapolated to the patients with extremely high FBG, HbA1c or type 1 diabetes. Qualitative changes in coagulation factors occur in diabetes and the fact that quantitative differences were not found does not mean that no effect is observed. Our study was based on assessment of the variables at a single time point only and we did not follow our patients to assess the incidence of thrombotic events. This analysis should be considered a hypothesis-generating study and a larger prospective study is needed to assess the actual links and the importance of hypoglycemia and clinical events in type 2 diabetes.

In summary, we demonstrated in patients with type 2 diabetes and high cardiovascular risk that a J-shaped relationship appears to exist between glucose levels and thrombin formation as well as fibrin clot structure parameters (density of plasma fibrin network and its lysability). Interestingly, we have found the alterations in fibrin clot phenotype and the increase in thrombin generation were even more pronounced that in patients with hyperglycemia. Given a similar relationship between FBG and cardiovascular mortality in epidemiological studies, we might hypothesize that repeated periods of asymptomatic low glycemia in type 2 diabetes contributes to a prolonged prothrombotic state, leading to increased mortality following hypoglycemia. These findings may enhance our understanding of the complex mechanisms behind elevated cardiovascular risk associated with type 2 diabetes.

## Abbreviations

ΔAbs_max_: maximum absorbance of fibrin gel at 405 nm determined by using turbidimetry
sCD40L: soluble CD_40_ ligand
CV: cardiovascular
D-D_max_: maximum D-dimer levels in the lysis assay
D-D_rate_: maximum rate of increase in D-dimer levels in the lysis assay
FBG: fasting blood glucose
HbA1c: glycated hemoglobin
IQR: interquartile range
K_s_: permeability coefficient
MI: myocardial infarction
PAI-1: plasminogen activator inhibitor-1
PF4: platelet factor 4
t_50%_: half-lysis time
TAFI: thrombin-activatable fibrinolysis inhibitor
